# Perovskite metasurfaces with large superstructural chirality

**DOI:** 10.1038/s41467-022-29253-0

**Published:** 2022-03-23

**Authors:** Guankui Long, Giorgio Adamo, Jingyi Tian, Maciej Klein, Harish N. S. Krishnamoorthy, Elena Feltri, Hebin Wang, Cesare Soci

**Affiliations:** 1 Centre for Disruptive Photonic Technologies, The Photonics Institute, Nanyang Technological University, 21 Nanyang Link, Singapore, 637371 Singapore; 2 School of Materials Science and Engineering, National Institute for Advanced Materials, Nankai University, 300350 Tianjin, China; 3 Division of Physics and Applied Physics, School of Physical and Mathematical Sciences, Nanyang Technological University, 21 Nanyang Link, Singapore, 637371 Singapore; 4 Department of Physics, Politecnico di Milano, Piazza Leonardo da Vinci 32, 20133 Milano, Italy

**Keywords:** Metamaterials, Metamaterials, Sub-wavelength optics

## Abstract

Recent attempts to synthesize hybrid perovskites with large chirality have been hampered by large size mismatch and weak interaction between their structure and the wavelength of light. Here we adopt a planar nanostructure design to overcome these limitations and realize all-dielectric perovskite metasurfaces with giant superstructural chirality. We identify a direct spectral correspondence between the near- and the far- field chirality, and tune the electric and magnetic multipole moments of the resonant chiral metamolecules to obtain large anisotropy factor of 0.49 and circular dichroism of 6350 mdeg. Simulations show that larger area metasurfaces could yield even higher optical activity, approaching the theoretical limits. Our results clearly demonstrate the advantages of nanostructrure engineering for the implementation of perovskite chiral photonic, optoelectronic, and spintronic devices.

## Abstract (web summary)

Though chiral hybrid organic-inorganic perovskites are attractive for next-generation optoelectronics, imparting strong chirality through chemical synthesis has proved challenging. Here, the authors report all-dielectric perovskite metasurfaces with giant superstructural chirality via planar nanostructuring.

## Introduction

**Fig. 1:**
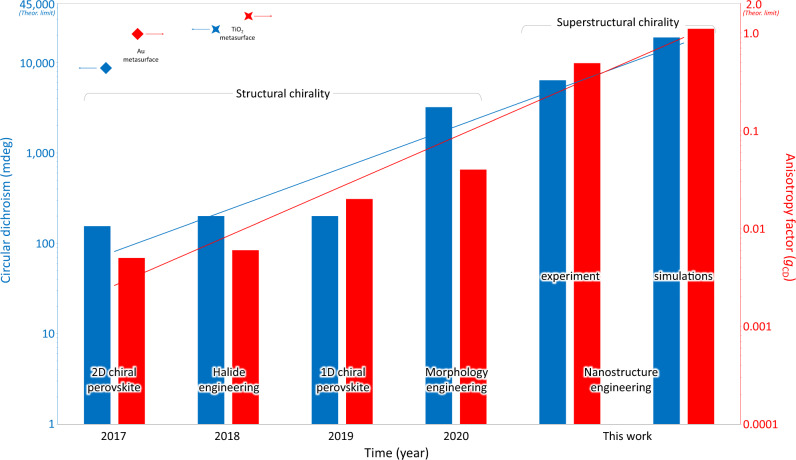
Approaching theoretical limits with superstructural chirality. In 2017, Moon et al. first investigated the chiroptical properties of 2D chiral perovskite film, reporting CD of 155 mdeg and *g*_CD_ of 0.005^14^. In 2018, Sargent et al. achieved CD of 200 mdeg and *g*_CD_ of 0.006 in a 2D bromide chiral perovskite film. In 2019, Tang et al. reported circularly polarized photodetectors based on a 1D chiral perovskite film with CD of 200 mdeg and *g*_CD_ of 0.02^19^. In 2020, Miyasaka et al. reported an encouraging CD of 3200 mdeg and *g*_CD_ of 0.04 after morphology optimization of the 1D chiral perovskite film^25^. Switching from structural to superstructural chirality, this work reports high CD of 6350 mdeg and large *g*_CD_ of 0.49 in a perovskite chiral metasurface obtained by nanostructure engineering. Numerical simulations predict CD of 18900 mdeg and *g*_CD_ of 1.1 in large area samples, coming closer to the theoretical limits of both parameters. The diamonds and 4-point stars indicate representative studies where metasurface with strong chirality were obtained using conventional optical materials like Au^40^ and TiO_2_^37^. The theoretical limits for CD ( ± 45000 mdeg) and g_CD_ (±2) are obtained using the formulas defined in the introduction and correspond to the case of one circular polarization (e.g. LCP) being fully absorbed and the other (e.g. RCP) fully transmitted.

Combining chirality with the remarkable optical^1–3^, electrical^4–7^, and spintronic properties^8–10^ of perovskites, chiral hybrid organic-inorganic perovskites are receiving considerable attention for applications in chiral optoelectronics^11–14^ and spintronics^15,16^, such as spin transport and control^15–18^, circularly polarized light (CPL) detection and emission^18–25^, second-harmonic generation^26–28^, and other linear and nonlinear chiroptical effects^29^. Nonetheless, due to the weak chirality transfer (limited degree of structural twisting) from chiral molecules to the perovskite framework, optical activity, and distinguishability of circularly polarization imparted on light passing through the sample is rather poor. Together with circular dichroism (CD), the degree of chirality of a medium can be quantified by the anisotropy factor (*g*_CD_). While CD measures the difference in absorption of circularly polarized light of opposite handedness^30^, which is proportional to the rotational strength of the medium, *g*_CD_ provides the degree of ellipticity a linearly polarized light acquires after traversing the chiral medium. The CD can therefore be defined as ${CD}={T}_{{{{{{\rm{RCP}}}}}}}-{T}_{{{{{{\rm{LCP}}}}}}}={A}_{{{{{{\rm{LCP}}}}}}}-{A}_{{{{{{\rm{RCP}}}}}}}$, if ${R}_{{{{{{\rm{RCP}}}}}}}={R}_{{{{{{\rm{LCP}}}}}}}$(Supplementary Note 1). Expressed in millidegrees, the CD, *θ* (mdeg), can alternatively be defined as: $\theta \left({{{{{\rm{mdeg}}}}}}\right)=\frac{180000}{\pi }{\arctan }(\frac{\sqrt{{T}_{{{{{{\rm{RCP}}}}}}}}-\sqrt{{T}_{{{{{{\rm{LCP}}}}}}}}}{\sqrt{{T}_{{{{{{\rm{RCP}}}}}}}}+\sqrt{{T}_{{{{{{\rm{LCP}}}}}}}}})$, while the anisotropy factor^31^ is given by ${g}_{{{{{{\rm{CD}}}}}}}=\frac{2({{lg}T}_{{{{{{\rm{RCP}}}}}}}-{{lg}T}_{{{{{{\rm{LCP}}}}}}})}{{{lg}T}_{{{{{{\rm{RCP}}}}}}}+{{lg}T}_{{{{{{\rm{LCP}}}}}}}}$ (Supplementary Note 2). As shown in Fig. 1, the highest anisotropy factor (*g*_CD_) of circularly polarized absorption reported to date in perovskites with structural chirality obtained by “bottom-up” synthesis is only 0.04^25^, far from the actual requirements of practical chiral optoelectronic and spintronic devices^11,32,33^. Further increase of chirality through molecular design is very challenging due to the negligible and hardly tunable magnetic transition dipole moment of chiral perovskites^23^. Thus, alternative strategies to produce chiral perovskite structures with strong optical activity^34^ are in high demand.

Nanostrucure engineering, through “top-down” fabrication of metasurfaces with chiral shapes or arrangements, has proven to be an effective strategy to impart strong superstructural chirality to achiral media^35–44^. This approach can deliver chiral metasurfaces with large optical activity using high-throughput screening of metamolecule designs by electromagnetic wave numerical simulations, thus saving significant time and reagent consumption needed in bottom-up synthetic approach. At the same time, the high and compositionally tunable refractive index (*n* > 1.9)^2,3^ of hybrid perovskites has enabled the realization of dielectric metasurfaces^45–48^ and photonic crystals with high-resolution structural colors^45,49,50^, enhanced photoluminescence^48,51–55^, anomalous reflection^38^, optical phase control^56^, third harmonic generation and three-photon luminecence^57^, and a variety of applications ranging from optical encryption^49^ and encoding^57^ to THz emission^58^, microlasers^49,51,59–61^, holography^50,56^, and ultrafast all-optical switching^62^. Here we show that a combination of metasurface design and perovskite nanostructuring^45,55^ can yield perovskite metasurfaces with giant superstructural chirality.

Specifically, we identify a previously unrecognized spectral correspondence between near- and far-field chirality, and use it to fine tune the electric and magnetic multipole moments of the resonant chiral metamolecules via high-throughput screening of the nanostructure design. We experimentally demonstrate a 20 µm × 20 µm planar-chiral perovskite metasurface with large anisotropy factor of *g*_CD_ = 0.49 and circular dichroism of CD = 6350 mdeg, and predict by simulations that *g*_CD_ = 1.11 and CD~18900 mdeg would be achievable in larger area metasurfaces. The methodological transition from chemical structure engineering to optical design of the metamolecules ensures continuity of the exponential improvement of perovskite circular dichroism and anisotropy factor, taking them closer to their theoretical limits (Fig. 1). We argue that superstrucural chirality opens new opportunities to couple optical chirality with compositional engineering, light-emission and detection, structural phase change, and spin-dependent transport properties of hybrid perovskites.

## Results

### Perovskite metasurfaces with high optical activity

For a proof-of-principle demonstration, we selected the methylammonium lead iodide perovskite, CH_3_NH_3_PbI_3_ (MAPbI_3_), a reliable high refractive index achiral platform for all-dielectric perovskite metasurfaces^45,56^. Thin perovskite films (~315 nm) were spin-cast on quartz substrates and used for fabricating the chiral metasurface. The dielectric functions of MAPbI_3_^45^ and quartz were used to design metasurfaces comprising of planar chiral metamolecules of mirror twist (Supplementary Fig. 1), with both unit cell size and period of 730 nm, targeting optical resonances in the low-loss, sub-band edge region of the perovskite.

**Fig. 2:**
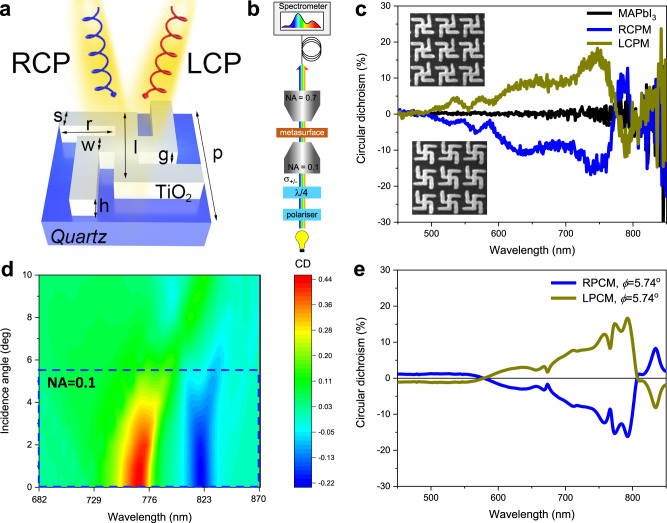
Giant circular dichroism of perovskite chiral metasurfaces. **a** Schematic of a left-handed perovskite chiral metamolecule on quartz substrate (*s* = 120 nm, *r* = 305 nm, *w* = 120 nm, *l* = 500 nm, *g* = 70 nm, *h* = 315 nm, *p* = 730 nm). **b** Micro-spectrometer setup for the measurement of circular dichroism on small area metasurfaces. **c** Experimental circular dichroism of unpatterned MAPbI_3_ film, right- and left-handed perovskite chiral metasurfaces (RPCM and LPCM), illuminated over a solid angle with NA of 0.1. The insets show scanning electron microscope images of 3×3 unit cells of LPCM and RPCM. **d** Color map of simulated circular dichroism of the LPCM as a function of wavelength and incidence angle *ϕ*. **e** Spectra of the simulated circular dichroism of the RPCM and LPCM for an incidence angle *ϕ* = 5.74^o^.

The representative unit of the perovskite chiral superstructure is shown in Fig. 2a. The gammadion metamolecule design was chosen based on the better performance compared to other possible designs (Supplementary Fig. 2). Optimal design parameters, bound by state-of-the-art nanofabrication tolerances and limits, were obtained by high-throughput superstructure screening via full wave electromagnetic Finite-Difference Time-Domain (FDTD) simulations (Supplementary Fig. 3), yielding *s* = 120 nm, *r* = 305 nm, *w* = 120 nm, *l* = 500 nm, *h* = 315 nm, and *p* = 730 nm. The FDTD simulations predict this resonant perovskite chiral metasurface design could generate a giant CD of 45% (Supplementary Fig. 4) at 767 nm under normal incidence, around the band edge of the MAPbI_3_, on-par with the best performing conventional planar dielectric nanostructures to date^37^. Two planar-chiral nanostructured perovskite metasurfaces of opposite handedness were carved on the MAPbI_3_ perovskite film by focused ion beam (FIB) milling, in arrays of about 20 µm × 20 µm area (Supplementary Fig. 5). The CD of perovskite chiral metasurface of both handedness and unpatterned MAPbI_3_ films was measured in transmission at quasi-normal incidence, using a microscope objective with NA = 0.1 (solid angle *ϕ* ~ 5.74°). The spectra were collected across the entire visible region, under both right- and left-handed circularly polarized light illumination, and detected using a grating spectrometer, as shown in Fig. 2b. Consistent with the simulation results (Supplementary Fig. 6), distinct peaks were observed in the experimental transmission spectra around 747 nm (Supplementary Fig. 7), leading to a remarkable circular dichroism experimental value of 16% (as shown in Fig. 2c). As expected, mirror symmetric left-handed (LPCM) and right-handed perovskite chiral metasurfaces (RPCM) exhibit opposite CD, whereas the unpatterned area of MAPbI_3_, or metasurfaces with achiral metamolecules (Supplementary Fig. 8), exhibit negligible CD through the entire visible region. When expressed in millidegrees, *θ* (mdeg), the CD of perovskite metasurface with superstructural chirality reaches a peak value of 6350 mdeg at 747 nm (Supplementary Fig. 9), an almost two-fold increase over the highest reported CD for “bottom-up” perovskite with structural chirality (3200 mdeg)^25^. At the same time. the *g*_CD_ = 0.49 of the perovskite metasurface with superstructural chirality (Supplementary Fig. 10) is almost 12 times higher than the best value for “bottom-up” perovskite with structural chirality reported so far (*g*_CD_ = 0.04^25^). Note that, while losses induced by the FIB milling process may affect the circular dichroism of the perovskite metasurfaces, it should be safe to neglect them in structures where the exposed regions are completely removed (see discussion in Supplementary Note 3 and Supplementary Fig. 18).

Angle-dependent numerical simulations allow examining the difference between predicted and experimentally measured CD spectra at incidence angles off the normal. The color map in Fig. 2d illustrates the dependence of CD spectra on incidence angle: the CD intensity decreases and broadens rapidly away from normal incidence, becoming almost featureless for *ϕ* > 7°. Within a *ϕ* ~ 5.74^o^ (corresponding to a numerical aperture of NA = 0.1) solid angle of incidence, the numerically simulated CD is in excellent agreement with the experimental results. This confirms that the role of optical losses induced by the FIB fabrication is negligible. Circular dichroism values well exceeding 40% (*θ* (mdeg)~18,900 mdeg) and anisotropy factor values higher than 1 (*g*_CD_ = 1.11), as shown in Supplementary Fig 11), are expected in large area devices illuminated at quasi-normal incidence, which could be realized by high-throughput nanofabrication techniques like nanoimprint lithography.

### Spectral correspondence of optical chirality and circular dichroism

**Fig. 3:**
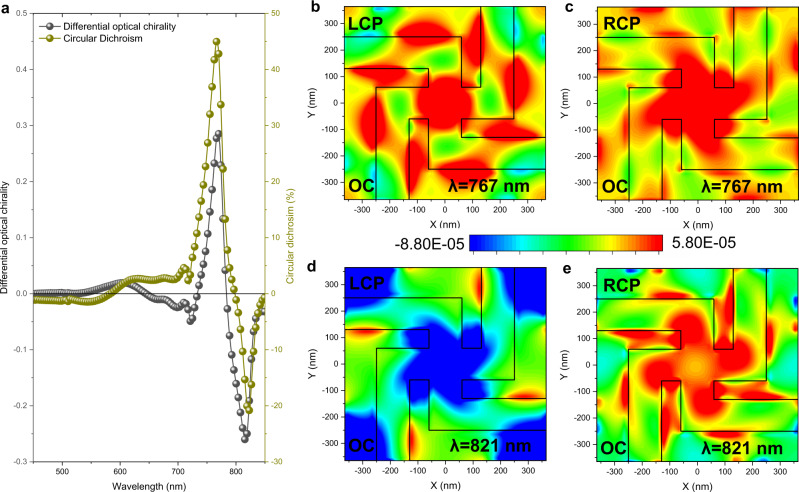
Optical chirality of perovskite chiral metasurfaces. **a** Simulated differential optical chirality (integrated over the bottom surface) as a function of wavelength for a left-handed perovskite chiral metasurface, closely following the behavior of circular dichroism. **b**–**e** Color maps showing dissimilar spatial distributions of optical chirality for a left-handed chiral gammadion metamolecule at **b** 767 nm under LCP illumination, **c** 767 nm under RCP illumination, **d** 821 nm under LCP illumination, and **e** 821 nm under RCP illumination. All maps refer to the escaping surface upon which the light is incident.

The origin of the giant CD generated by the perovskite chiral metasurfaces can be understood by investigating the near-field interaction of the metamolecules with the incident light and how this relates to the chiral response observed in the far-field. The chirality of the optical near-field can be gauged by the optical chirality, OC^63^, while the combination of electromagnetic multipoles can be employed to predict how the near-field mode distribution radiates into the far-field. OC is a time-even pseudoscalar, introduced as a measurement of field chirality, that describes the rate to which, at each point in space, electric and magnetic field vectors coil around a helical axis^64^. This is particularly interesting in the near-field of nanostructures where so-called superchiral fields^65^ are generated by the complex interaction with circularly polarized light. The analytical expression for the OC, proposed mathematically in 1964^66^, and recently correlated to the chiral asymmetry of the rate of excitation of a small chiral molecule^67^, can be approximated by

1 $${OC}=-\frac{{\varepsilon }_{0}w}{2}{Im}\left[{{{{{{\boldsymbol{E}}}}}}}^{* }\cdot {{{{{\boldsymbol{B}}}}}}\right],$$

where *ε*_0_ is the permittivity of free space, *w* is angular frequency, ***E*** and ***B*** are the complex amplitudes of the electric field and magnetic field, respectively. Simulated OCs induced by light waves of opposite helicity were integrated on the output surface of the perovskite chiral metasurface, and their difference was derived as a function of excitation wavelength. The differential OC and the numerically calculated CD reveal a remarkable spectral correspondence (Fig. 3a), thus providing a direct link between far- and near-field chirality. The near-field chiral interaction opens the possibility to tune the perovskite intrinsic optical properties such as Purcell enhancement leading to luminescence increase^45^ and lasing^68^ and modification of optical selection rules through the creation of virtual optical states^69^. Figure 3b–e show the optical chirality maps for a metamolecule of the left-handed chiral metasurface at the differential OC peak wavelengths, 767 nm and 821 nm, under both left- and right-handed circularly polarized incident light. As expected for gammadion metamolecules^63^, the optical chirality maps exhibit strong hotspots and distinct anti-clockwise twists at both wavelengths, yet with some notable differences. While the color maps for 767 nm-LCP (Fig. 3b) and RCP (Fig. 3c) incident light exhibit both positive OC, with different spatial distribution, the color maps for 821nm-LCP (Fig. 3d) and RCP (Fig. 3e) incident light have opposite OC values. These differences suggest that the strong CD of the perovskite metasurface at 767 nm and 821 nm shall be attributed to different combinations of electromagnetic modes of the metamolecule.

### Contribution of scattering multiples to circular dichroism

**Fig. 4:**
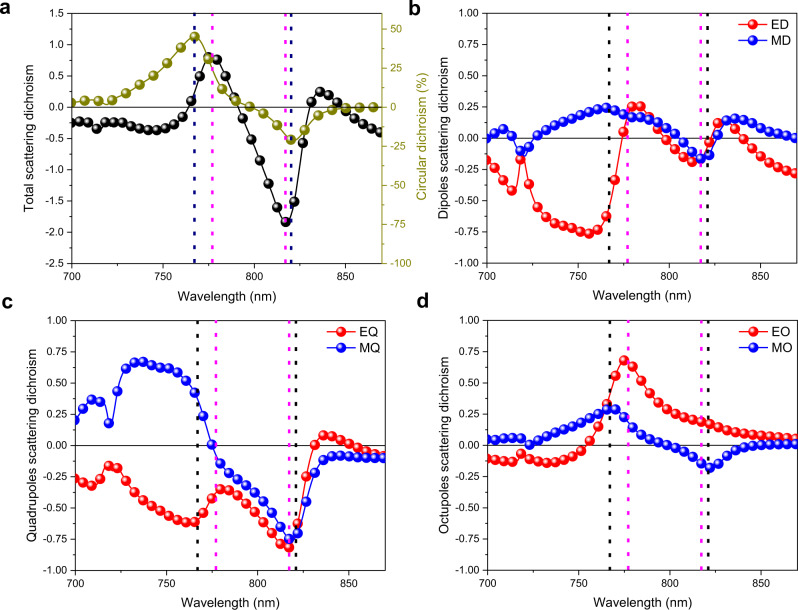
Scattering cross section dichroism of the electromagnetic multipoles in perovskite chiral metasurfaces. **a** Closely matching response of total scattering dichroism, ${{CD}}_{{C}_{S}}$ (black curve), and circular dichroism, CD (yellow curve), as functions of the wavelength. **b**–**d** Contribution of individual multipoles to the strong dichroism of the perovskite chiral metasurface: **b** electric (red curve) and magnetic (blue curve) dipole scattering dichroisms, ${{CD}}_{{C}_{{ED}}}$ and, ${{CD}}_{{C}_{{MD}}}$, as function of the wavelength; **c** electric (red curve) and magnetic (blue curve) quadrupole scattering dichroisms, ${{CD}}_{{C}_{{EQ}}}$ and, ${{CD}}_{{C}_{{MQ}}}$, as function of the wavelength; **d** electric (red curve) and magnetic (blue curve) octupole scattering dichroisms, ${{CD}}_{{C}_{{EO}}}$and, ${{CD}}_{{C}_{{MO}}}$, as function of the wavelength. The vertical black dotted lines indicate the peaks of the CD spectrum, while the pink dotted lines correspond to the peaks of the ${{CD}}_{{C}_{S}}$ spectrum.

Decomposition into multipoles of the metamolecule scattering cross sections, under both left- and right-handed circularly polarized illumination, allows identifying the electromagnetic modes responsible for the giant CD. The modes are expected to have components of electric and magnetic moments parallel to each other (i.e., resulting electric fields aligned perpendicularly), which induce polarization rotation and optical activity. This is a generalization of the so-called Rosenfeld criterion^30,70^, which requires a non-null cross-product of the net electric and magnetic dipole moments, ${{{{{\boldsymbol{p}}}}}}{{{{{\boldsymbol{\cdot }}}}}}{{{{{\boldsymbol{m}}}}}}\ne 0$, as condition to observe chiro-optical activity (see Supplementary Figs. 12–15). The *total scattering dichroism* can be defined as ${{CD}}_{{C}_{S}}=({C}_{{S}_{L}}-{C}_{{S}_{R}})$, where ${C}_{{S}_{L}}$and ${C}_{{S}_{R}}$ are the total scattering cross sections of a metamolecule of chosen handedness under left-handed and right-handed circularly polarized light, respectively. The spectral response of ${{CD}}_{{C}_{S}}$ follows closely that of CD, with a distinct peak around 767 nm and dip around 821 nm (Fig. 4a). The small discrepancy between the curves can be attributed to the different definitions of total scattering cross section and transmission (see Methods). Since the total scattering cross-section is proportional to the sum of scattering intensities of the multipoles, ${C}_{S}\propto {\sum }_{i}{C}_{{S}_{i}}$ (see Methods for exact formulation), the total scattering dichroism can be expressed as the sum of individual *multipoles scattering dichroisms*, ${{CD}}_{{C}_{S}}={\sum }_{i}{{CD}}_{{C}_{E}}\left(i\right)+{{CD}}_{{C}_{M}}\left(i\right)$, where ${{CD}}_{{C}_{E/M}}\left(i\right)$ is the dichroism of the scattering cross section of the electric/magnetic multipole of order *i*. This makes it possible to quantify the contribution of each multipole to the dichroism.

The multipoles mainly responsible for the chiral response of the perovskite gammadion metamolecules are the electric and magnetic dipoles (ED, MD), quadrupoles (EQ, MQ), and octupoles (EO, MO); their scattering dichroisms are shown in Fig. 4b–d. Other modes such as electric and magnetic hexadecapoles (EH, MH) are negligible (Supplementary Fig. 16). It is worth noting that the spectral distribution of the CD does not correlate with high-intensity multipoles, rather with their individual scattering dichroism. For example, while the magnetic dipole is the strongest scattering multipole at wavelengths longer than 800 nm (Supplementary Fig. 17), the high circular dichroism at 821 nm is mainly caused by the difference in scattering strengths of the electric and magnetic quadrupoles for LCP and RCP excitation (Fig. 4c), not of the magnetic dipoles. On the other hand, the chiral response of the strong circular dichroism at shorter wavelengths (767 nm) stems from the cooperative effect of all electric and magnetic multipoles up to the third order (Fig. 4b–d).

## Discussion

We demonstrated a dielectric perovskite metasurface with giant chirality. Circular dichroism of 6350 mdeg and anisotropy factor of 0.49 were achieved experimentally, with simulations showing that larger area metasurfaces could yield anisotropy factor of 1.11 and circular dichroism of ~18900 mdeg, close to their theoretical limits. The remarkably strong chiroptical behavior results from the fine tuning of geometrical parameters and electromagnetic multipole moments competition, following the newly recognized spectral correspondence between near- and far-field chirality. These results show that the change in methodology, from chemical to superstructural engineering can perpetuate the exponential improvement of perovskite circular dichroism and anisotropy factor. The nanostructure engineering approach, aided by high-throughput screening of metamolecule shapes and parameters via electromagnetic wave numerical simulations, can extend this paradigm to the entire visible and near infrared spectrum, while saving considerable time and use of reagents needed in bottom-up synthesis. Furthermore, as circular dichroism may also be imprinted onto the light emitted by perovskite metasurfaces, this approach may open the way to new types of polarization-encoding light emitting devices. Overall, the concurrence of high refractive index, strong optical activity, excellent radiative properties, and large area manufacturability, makes hybrid perovskite metasurfaces a truly unique platform for chiral photonic, optoelectronic and spintronic devices.

## Methods

### Thin-film fabrication

MAPbI_3_ solution was prepared by predesigned amount of PbI_2_ (165.96 mg, TCI, 99.99%) and methylammonium iodide (57.2 mg, Dyesol) in stoichiometric ratio dissolved in 0.3 mL DMF (anhydrous, Sigma Aldrich). The 1.2 M solution was stirred overnight at room temperature in a N_2_ filled glovebox, then filtered by a polyvinylidene fluoride (PVDF) syringe filter (0.45 μm) and left stirring at 100 °C for one hour before spin coating. The resulting solution was then spin coated with a one-step process at 3500 rpm for 30 s onto pre-cleaned 1 × 1 cm^2^ quartz substrates in N_2_ atmosphere. After 6 s from the beginning of the spin coating process, 500 μL of toluene (anhydrous, Sigma Aldrich) was poured onto the spinning sample. The resulting films were then annealed at 100 °C for 15 min to improve crystallization. The thickness of the perovskite film is *ca*. 315 nm.

### Metasurface fabrication

The 20 × 20 µm arrays of gammadion metasurfaces, of opposite handedness, were patterned on the perovskite film, spin-coated on a quartz substrate, with a Helios 650 NanoLab Focused Ion Beam system, using a nominal beam current <5 pA.

### FDTD simulations

The circular dichroism of the perovskite chiral metasurface was simulated by finite difference time-domain method (Lumerical FDTD Solutions). The representative unit of the perovskite chiral superstructure is shown in Fig. 2a, with design parameters *s* = 120 nm, *r* = 305 nm, *w* = 120 nm, *l* = 500 nm, *h* = 315 nm and *p* = 730 nm, respectively. Right (Left) circularly polarized light is incident along z-axis. Perfect Matched Layers (PML) are used in the propagation direction of the incident light and periodic boundary conditions are used in the directions normal to the propagation direction. The optical chirality is calculated according to Eq. 1 based on the electromagnetic field at the bottom surface of the superstructure. For the angle-dependent CD, the Bloch boundary conditions are applied to the directions normal to the light propagation direction.

### Circular dichroism spectra

The circular dichroism spectra were obtained by measuring the optical transmission of right- and left-handed chiral metasurfaces, under right- and left-handed circularly polarized illumination, in a Nikon inverted optical microscope, equipped with a halogen lamp (as shown in Fig. 2b). The polarizations of the incident light were prepared by sending the light through a polarizer and a broadband λ/4 waveplate (Thorlabs AQWP05M-580) with nearly achromatic transmission, 0.95 < *T* < 0.98, and retardance, 0.24 < *ρ* < 0.26, in the spectral region investigated. The light was focused on the sample using a Nikon LWD Achromat Condenser, with 10 mm working distance and adjustable NA. To ensure light collection from a spot smaller than the metasurface array, we used a Nikon ×100 objective with 0.7 NA and a multimode optical fiber, acting as pinhole. The spectra were recorded using an Acton SpectraPro 2300i monochromator and spectrograph.

### Multipole decomposition

To identify the electromagnetic modes responsible for the giant CD, a multipole decomposition of the scattering cross sections of a single gammadion metamolecule in the arrays under both left-handed and right-handed circularly polarized illumination is first conducted using FDTD^71^. The origin of the coordinate of multipole decomposition is chosen to be at the center of the metamolecule to minimize unnecessary high-order multipolar contributions.

The electric field *E*(r) inside the metamolecule is extracted from the simulation to define the polarization current $J\left({{{{{\boldsymbol{r}}}}}}\right)=-{{{{{\rm{i}}}}}}{{{{{\rm{\omega }}}}}}{\varepsilon }_{0}[{\varepsilon }_{r}\left({{{{{\boldsymbol{r}}}}}}\right)-1]E({{{{{\boldsymbol{r}}}}}})$. The electric ${a}_{E}\left(l,m\right)$ and magnetic ${a}_{m}\left(l,m\right)$ spherical multipole coefficients can then be calculated as follows^71^,

2 $${a}_{E}\left(l,m\right) =	\;\frac{{(-i)}^{l-1}{k}^{2}\eta {O}_{{lm}}}{{E}_{0}{[\pi (2l+1)]}^{1/2}}\int {{\exp }}\left(-{im}\varphi \right)\{\left[{\varPsi }_{l}\left({kr}\right)+{\varPsi }_{l}^{{\prime} {\prime} }\left({kr}\right)\right]{P}_{l}^{m}\left({\cos }\theta \right)\hat{r}\cdot J\left(r\right)\\ 	+\frac{{\varPsi }_{l}^{{\prime} }\left({kr}\right)}{{kr}}[{\tau }_{{lm}}(\theta )\hat{\theta }\cdot J\left(r\right)-{{{{{\rm{i}}}}}}{{{{{{\rm{\pi }}}}}}}_{{lm}}(\theta )\hat{\phi }\cdot J\left(r\right)]\}{d}^{3}r$$

3 $${a}_{m}\left(l,m\right)=\frac{{(-i)}^{l+1}{k}^{2}\eta {O}_{{lm}}}{{E}_{0}{[\pi (2l+1)]}^{1/2}}\int {{\exp }}\left(-{im}\varphi \right){j}_{l}\left({kr}\right)[{\tau }_{{lm}}(\theta )\hat{\phi }\cdot J\left(r\right)+{{{{{\rm{i}}}}}}{{{{{{\rm{\pi }}}}}}}_{{lm}}(\theta )\hat{\theta }\cdot J\left(r\right)]{d}^{3}r$$

where *η* is the impedance of free space; ${\varPsi }_{l}\left({kr}\right)={kr}{j}_{l}\left({kr}\right)$ are the Riccati-Bessel functions and ${\varPsi }_{l}^{{\prime} }\left({kr}\right)$ and ${\varPsi }_{l}^{{\prime} {\prime} }\left({kr}\right)$ are their first and second derivatives with respect to the argument *kr*; ${P}_{l}^{m}$ are the associated Legendre polynomials; ${O}_{{lm}}=\frac{1}{{[l(l+1)]}^{1/2}}{[\frac{2l+1}{4\pi }\frac{\left(l-m\right)!}{\left(l+m\right)!}]}^{1/2}$; ${\tau }_{{lm}}\left(\theta \right)=\frac{d}{d\theta }{P}_{l}^{m}({\cos }\theta )$; ${\pi }_{{lm}}\left(\theta \right)=\frac{m}{{\sin }\theta }{P}_{l}^{m}({\cos }\theta )$.

The total scattering cross section *C*_s_ of the metamolecule can be written as the sum of contributions from these multipoles,

4 $${C}_{s}=\frac{\pi }{{k}^{2}}\mathop{\sum }\limits_{l=1}^{{{\infty }}}\mathop{\sum }\limits_{m=-l}^{l}\left(2l+1\right)[{\left|{a}_{E}(l,m)\right|}^{2}+{\left|{a}_{M}(l,m)\right|}^{2}]$$

These equations allow to calculate the scattering cross sections from spherical multipoles of arbitrarily order *l*.

## Supplementary information

Supplementary Information

## Data Availability

The authors declare that all data supporting the findings of this study are available within this article and its supplementary information and are openly available in NTU research data repository DR-NTU (Data) at 10.21979/N9/N43DZX. Additional data related to this paper may be requested from the authors.
