# Supplementary material for: Perovskite metasurfaces with large superstructural chirality
Source: Nat Commun. 2022 Mar 23;13:1551. doi: 10.1038/s41467-022-29253-0 (PMC8943210; doi:10.1038/s41467-022-29253-0)
Supplement: Supplementary file 1 — Supplementary Information [file 41467_2022_29253_MOESM1_ESM.pdf]

Supplementary Information for

Perovskite metasurfaces with large superstructural chirality

Guankui Long[1,2†], Giorgio Adamo[1,†], Jingyi Tian[1], Maciej Klein[1,3], Harish N. S. Krishnamoorthy[1], Elena Feltri[1,4], Hebin Wang[2], and Cesare Soci[1,3]*

[1]Centre for Disruptive Photonic Technologies, The Photonics Institute, Nanyang Technological University, 21 Nanyang Link, Singapore 637371

[2]School of Materials Science and Engineering, National Institute for Advanced Materials, Nankai University, 300350, Tianjin, China

[3]Division of Physics and Applied Physics, School of Physical and Mathematical Sciences, Nanyang Technological University, 21 Nanyang Link, Singapore 637371

[4]Department of Physics, Politecnico di Milano, Piazza Leonardo da Vinci 32, 20133 Milano, Italy

*Correspondence to: csoci@ntu.edu.sg

†These authors contributed equally to this work.

Inventory

Supplementary Figure 1 | Dielectric functions of perovskite films and substrates........3
Supplementary Figure 2 | CD simulations for various shapes of $MAPbI_3$ chiral metamolecules...4
Supplementary Figure 3 | Optimization of CD in gammadion metasurface..................5
Supplementary Figure 4 | Simulated CD of perovskite chiral metasurfaces 6
Supplementary Figure 5 | Electron micrographs of perovskite chiral metasurfaces......7
Supplementary Figure 6 | Optical properties of perovskite chiral metasurfaces 8
Supplementary Figure 7 | Optical transmission of unpatterned and patterned perovskite films..9
Supplementary Figure 8 | SEM images and CD of the achiral metasurface.................10
Supplementary Figure 9 | Circular dichroism of unpatterned and patterned perovskite films 11
Supplementary Figure 10 | Anisotropy factor of unpatterned and patterned perovskite films 12
Supplementary Figure 11 | Simulated circular dichroism and anisotropy factor of perovskite chiral metasurfaces...13
Supplementary Figure 12 | Electromagnetic field maps of perovskite chiral metasurfaces at 767 nm...14
Supplementary Figure 13 | Electromagnetic field maps of perovskite chiral metasurfaces at 821 nm...15
Supplementary Figure 14 | Electromagnetic field maps of perovskite chiral metasurfaces at 767 nm...16
Supplementary Figure 15 | Electromagnetic field maps of perovskite chiral metasurfaces at 821 nm...17
Supplementary Figure 16 | Hexadecapoles scattering dichroism 18
Supplementary Figure 17 | Electromagnetic multipoles of the left-handed perovskite chiral metasurface 19
Supplementary Note 1...20
Supplementary Note 2...21
Supplementary Note 3...22
Supplementary Figure 18 | Optical microscope images in reflection and transmission mode of pristine $MAPbI_3$ film on quartz with FIB exposed areas...............................23

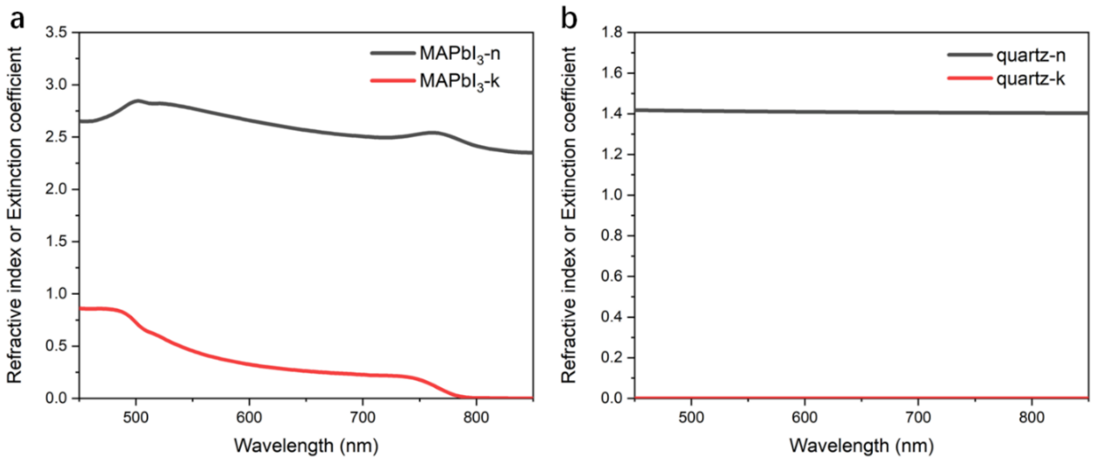

Supplementary Figure 1 | Dielectric functions of perovskite films and substrates. Experimentally determined dielectric functions of (a) $MAPbI_3$ (Refs [45], [55] in the main manuscript) and (b) quartz substrate.

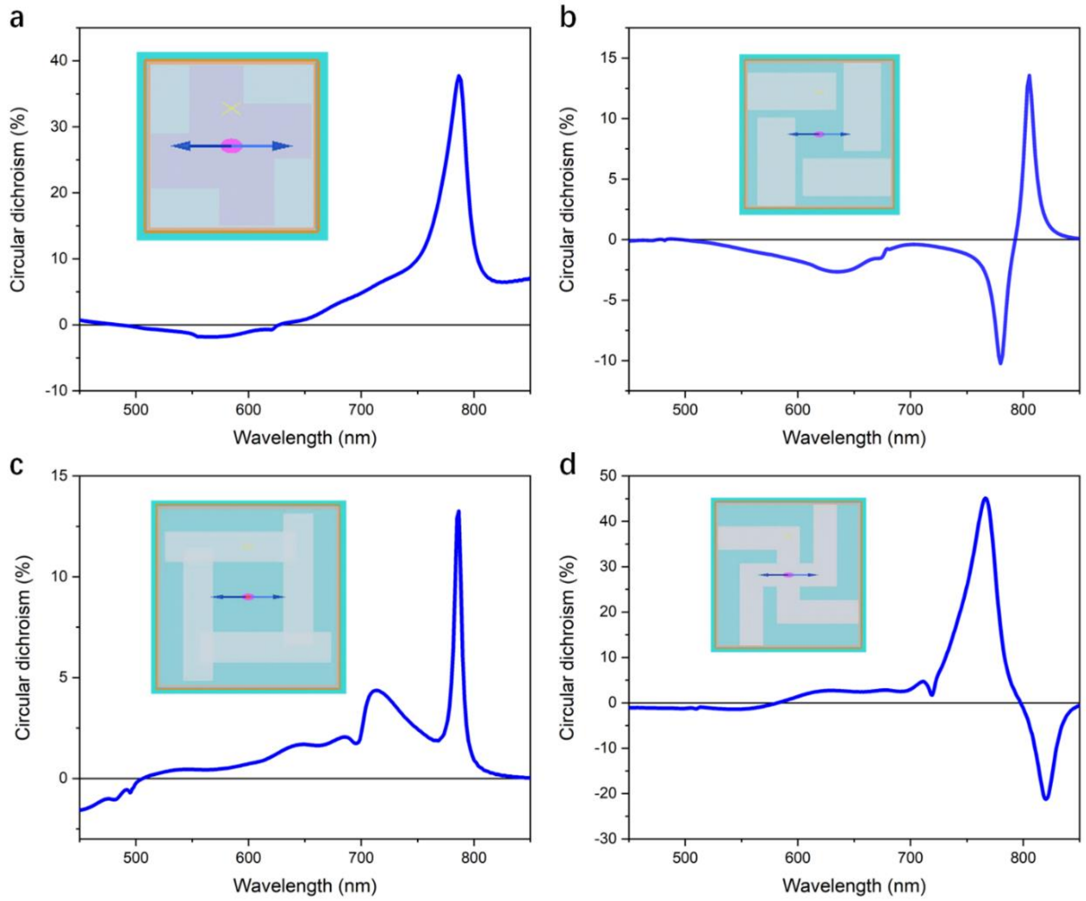

Supplementary Figure 2 | CD simulations for various shapes of $MAPbI_3$ chiral metamolecules. Chiral metamolecule designs considered for optimization of CD (a-c). The gammadion metamolecule design (d) was chosen based on the higher expected CD.

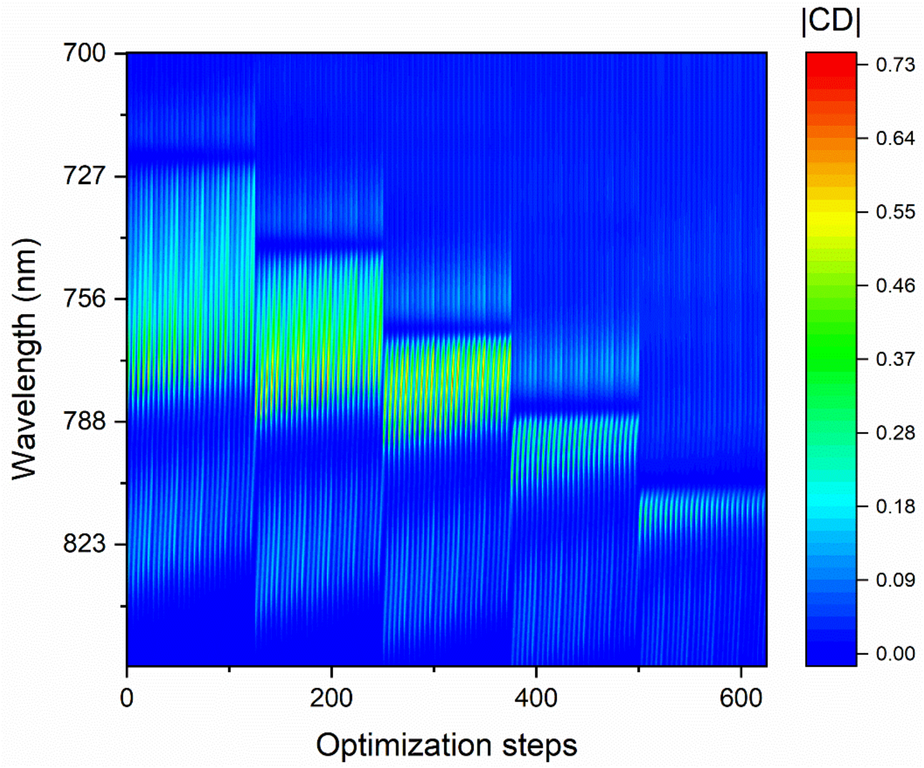

Supplementary Figure 3 | Optimization of CD in gammadion metasurface. The improvement of CD of the gammadion metasurface (Supplementary Fig. 2d) upon sequential variation of the geometrical parameters listed in Fig. 2a is shown in color scale.

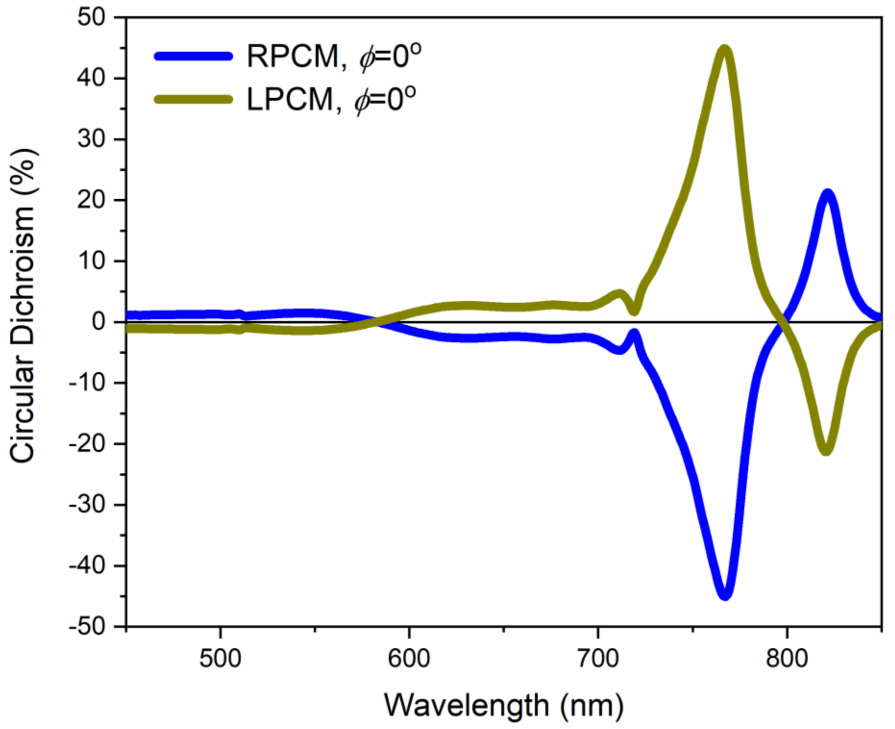

Supplementary Figure 4 | Simulated CD of gammadion perovskite chiral metasurfaces. Right-handed perovskite chiral metasurface (RPCM, blue line) and and left-handed perovskite chiral metasurface (LPCM, brown line) calculated at normal incidence (ϕ = 0°).

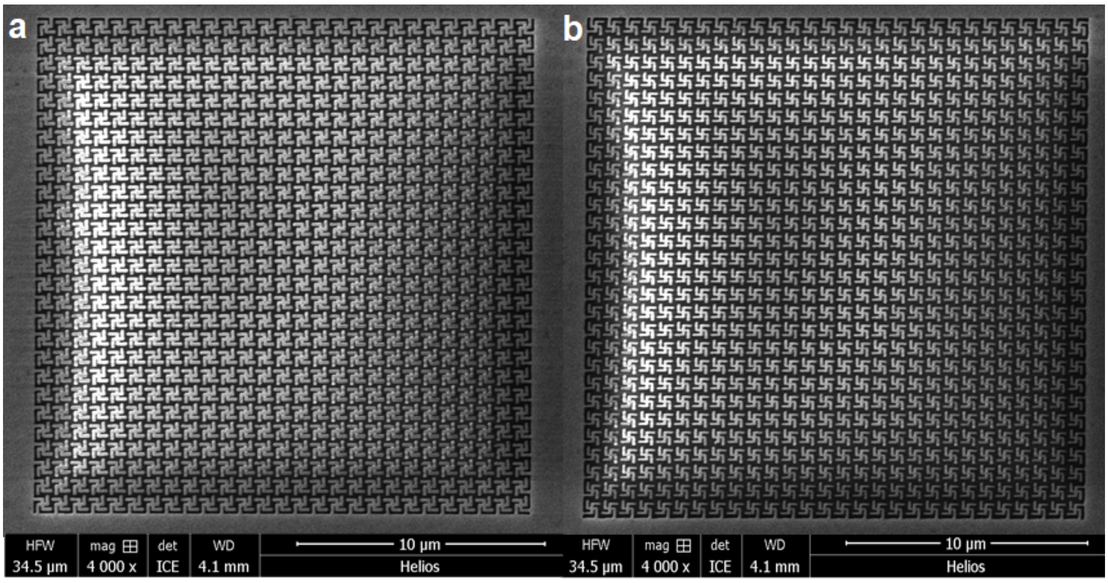

Supplementary Figure 5 | Electron micrographs of perovskite chiral metasurfaces. Scanning electron microscope images of the 20 µm × 20 µm arrays of LPCM (a) and RPCM (b).

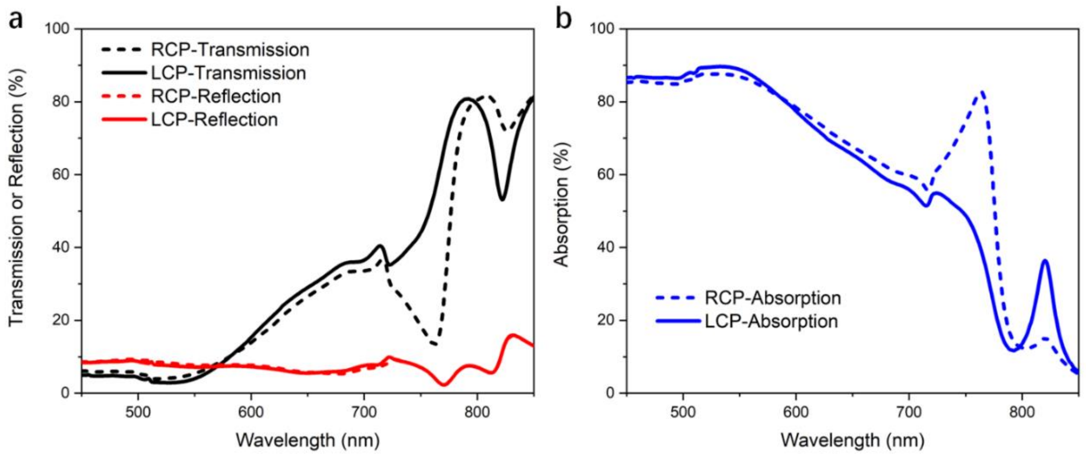

Supplementary Figure 6 | Optical properties of perovskite chiral metasurfaces. Simulated spectra of (a) reflection (red), transmission (black) and (b) absorption (blue) for a left-handed perovskite chiral metasurface under LCP (solid curves) and RCP (dashed curves) illumination.

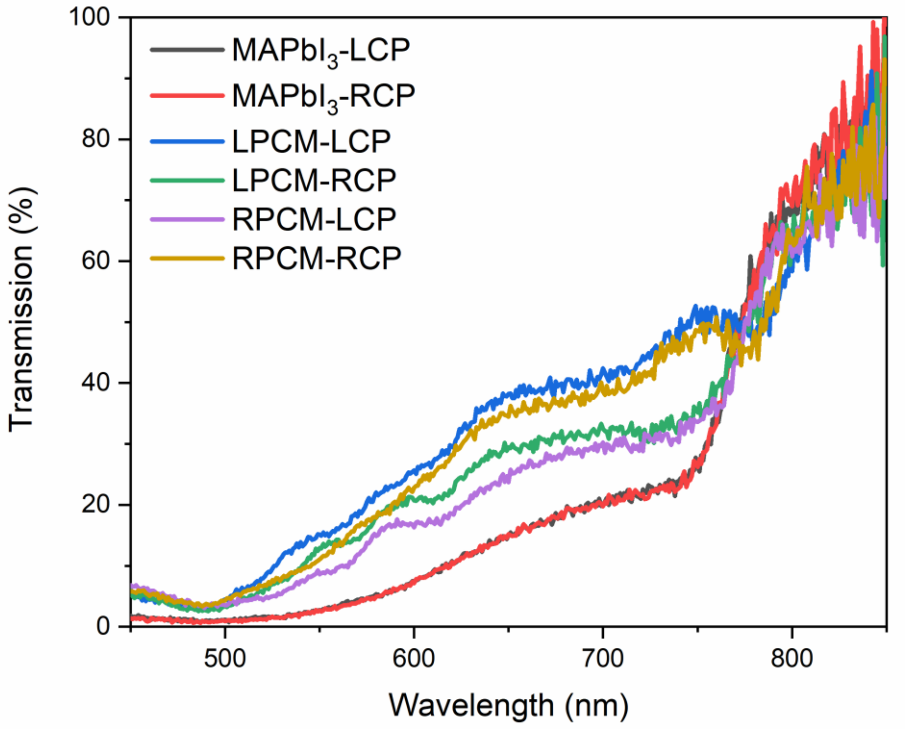

Supplementary Figure 7 | Optical transmission of unpatterned and patterned perovskite films. Experimental transmission spectra of unpatterned $MAPbI_3$ film, left- and right-handed perovskite chiral metasurfaces (RPCM and LPCM) under left-handed and right-handed circularly polarized light (illumination NA = 0.1).

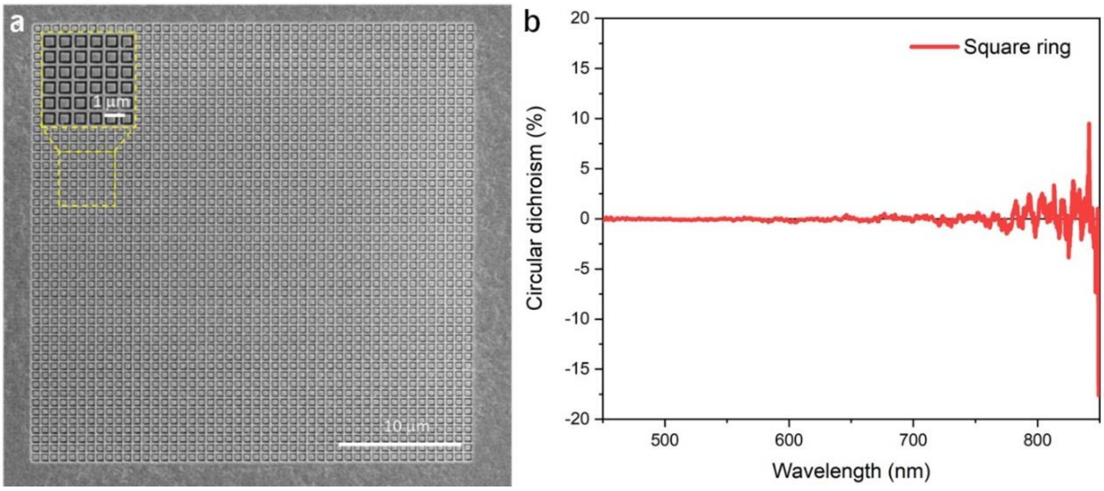

Supplementary Figure 8 | SEM images and CD of the achiral metasurface. a. SEM image of a square ring metasurface; b. CD of the square ring metasurface shows negligible CD through the entire visible region.

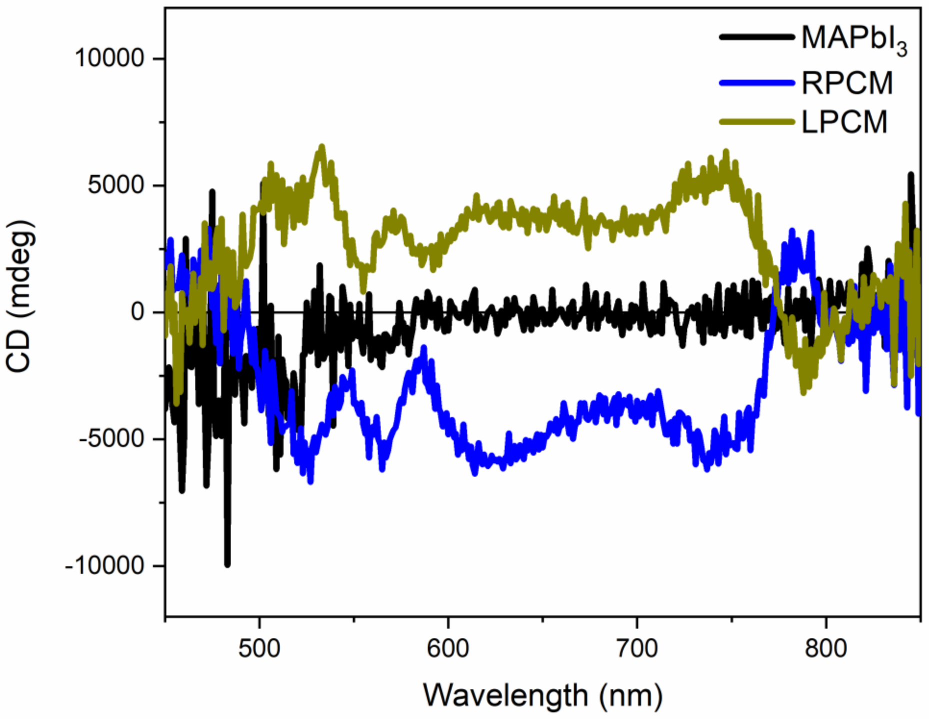

Supplementary Figure 9 | Circular dichroism of unpatterned and patterned perovskite films. Experimental circular dichroism (in mdeg) of unpatterned $MAPbI_3$ film, right- and left-handed perovskite chiral metasurfaces (RPCM and LPCM), illuminated with NA = 0.1.

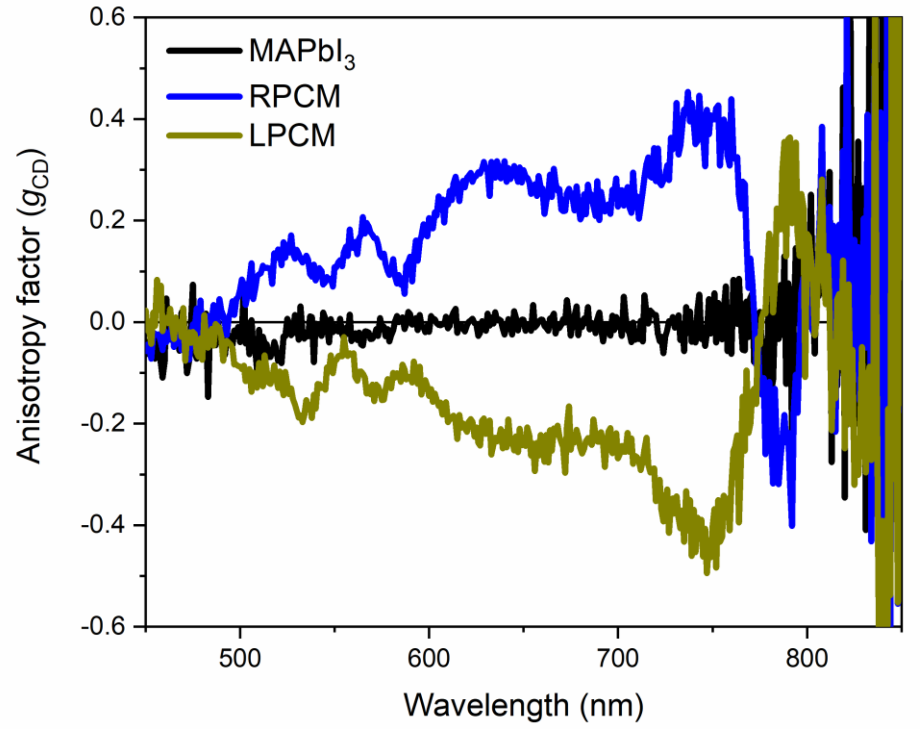

Supplementary Figure 10 | Anisotropy factor of unpatterned and patterned perovskite films. Experimental g_{CD} of unpatterned $MAPbI_3$ film, right- and left-handed perovskite chiral metasurfaces (RPCM and LPCM), illuminated with NA = 0.1.

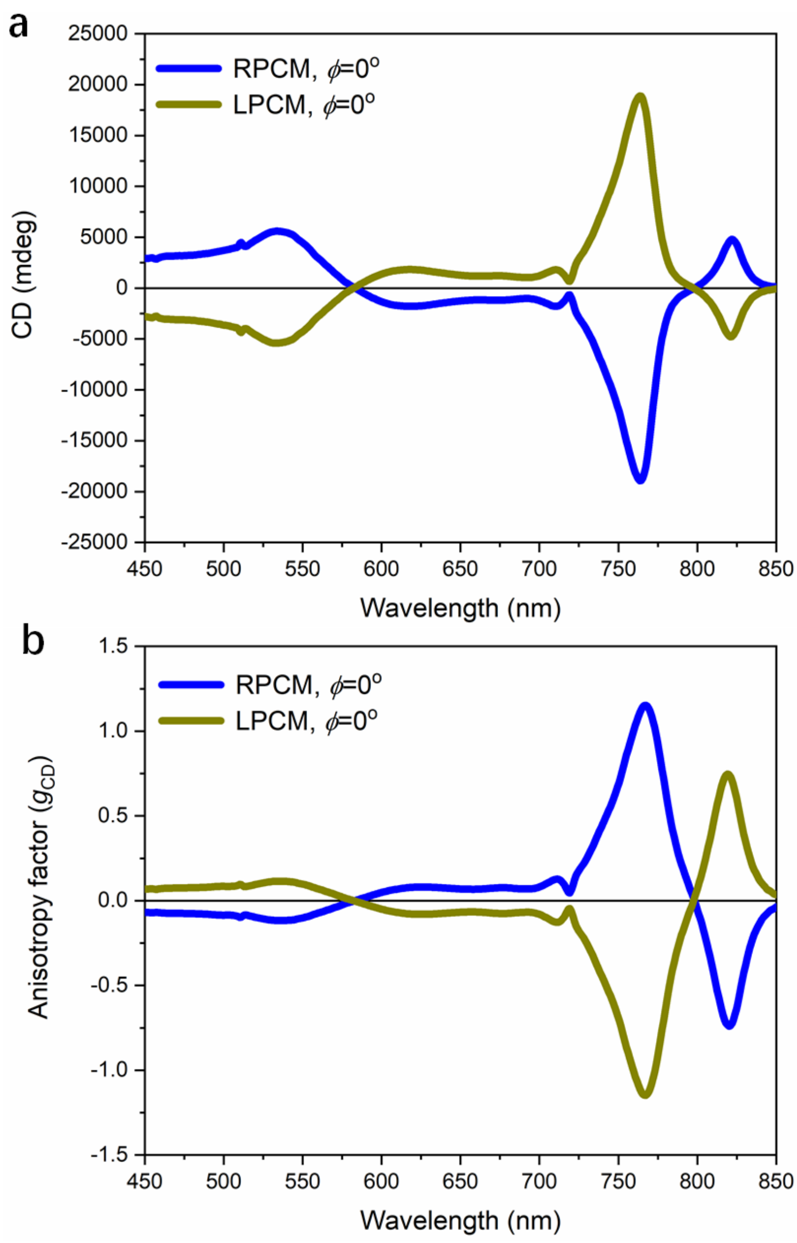

Supplementary Figure 11 | Simulated circular dichroism and anisotropy factor of perovskite chiral metasurfaces. Circular dichroism (a) and g_{CD} (b) of right- and left-handed perovskite chiral metasurfaces (RPCM and LPCM) under normal incidence (NA=0).

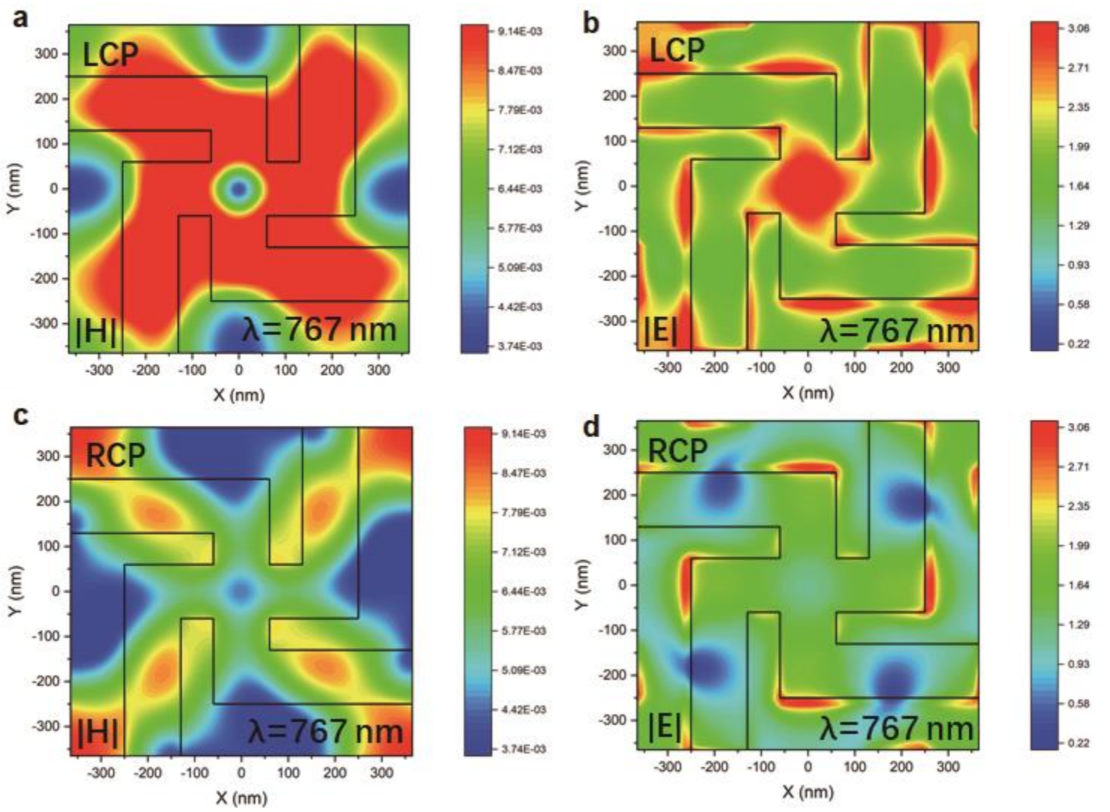

Supplementary Figure 12 | Electromagnetic field maps of perovskite chiral metasurfaces. **a-b**, Magnetic and electrical field amplitude maps for the left-handed perovskite gammadion metasurface at 767 nm under LCP illumination; **c-d**, Magnetic field and electrical field amplitude maps for the left-handed perovskite gammadion metasurface at 767 nm RCP illumination. All maps refer to the bottom surface of the structure upon which the light is escaping.

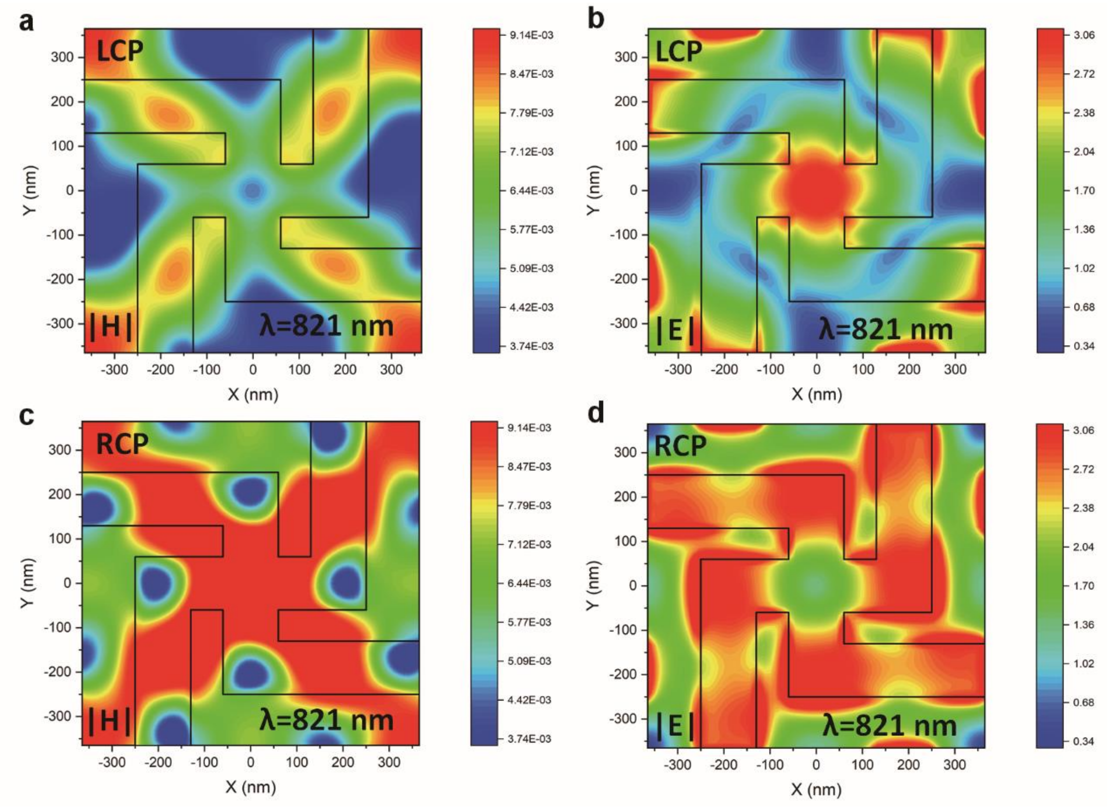

Supplementary Figure 13 | Electromagnetic field maps of perovskite chiral metasurfaces. **a-b**, Magnetic and electrical field amplitude maps for the left-handed perovskite gammadion metasurface at 821 nm under LCP illumination; **c-d**, Magnetic field and electrical field amplitude maps for the left-handed perovskite gammadion metasurface at 821 nm RCP illumination. All maps refer to the bottom surface of the structure upon which the light is escaping.

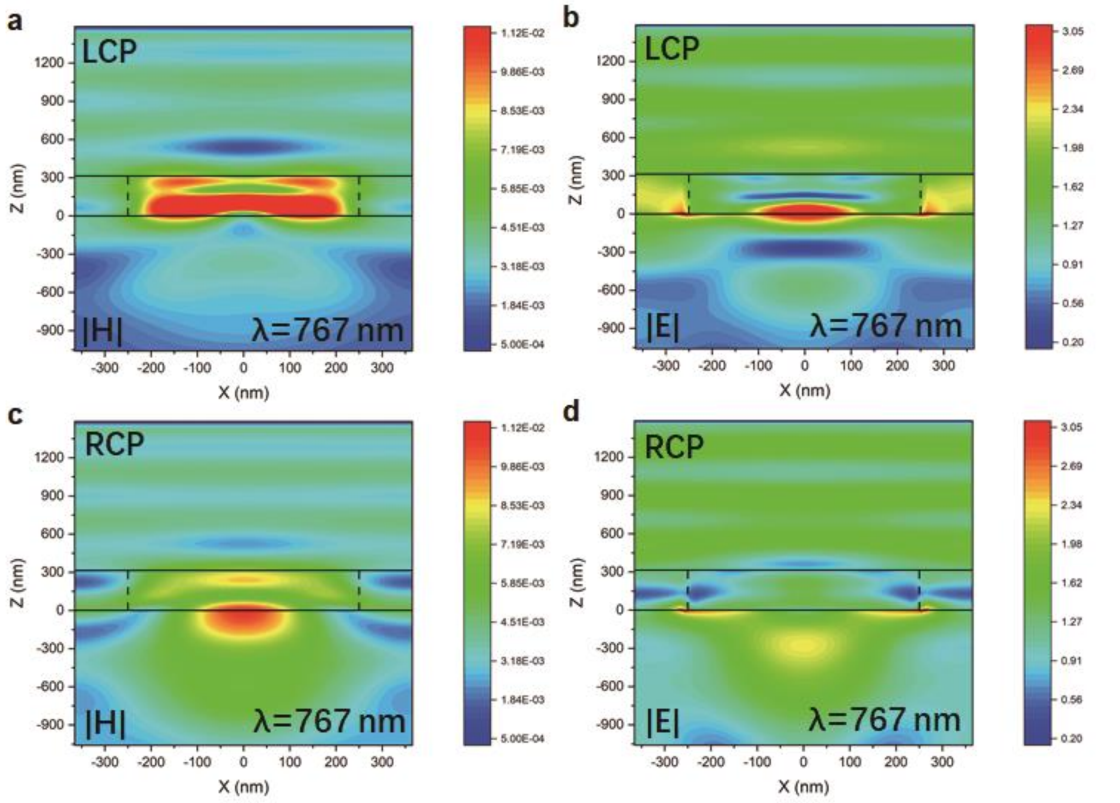

Supplementary Figure 14 | Electromagnetic field maps of perovskite chiral metasurfaces. **a-b**, Magnetic and electrical field amplitude maps for the left-handed perovskite gammadion metasurface at 767 nm under LCP illumination; **c-d**, Magnetic field and electrical field amplitude maps for the left-handed perovskite gammadion metasurface at 767 nm RCP illumination. All maps refer to the vertical cut through the middle of the chiral structure (x–z plane).

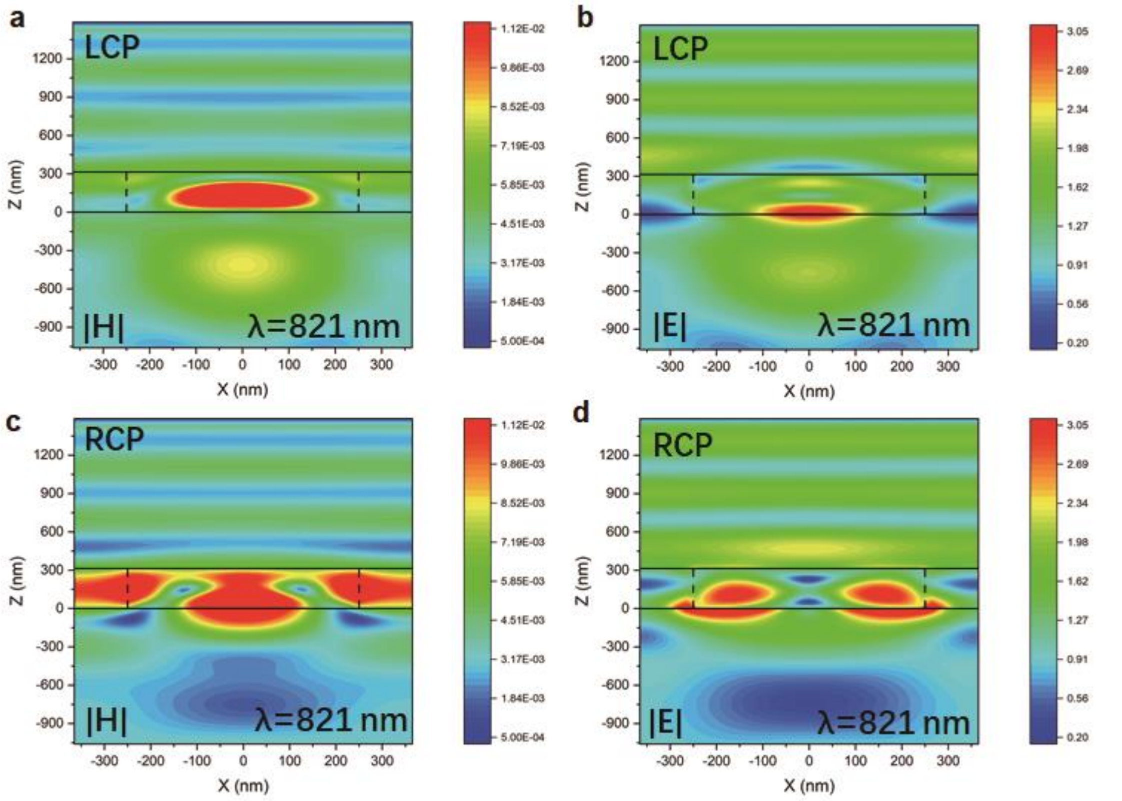

Supplementary Figure 15 | Electromagnetic field maps of perovskite chiral metasurfaces. **a-b**, Magnetic and electrical field amplitude maps for the left-handed perovskite gammadion metasurface at 821 nm under LCP illumination; **c-d**, Magnetic field and electrical field amplitude maps for the left-handed perovskite gammadion metasurface at 821 nm RCP illumination. All maps refer to the vertical cut through the middle of the chiral structure (x–z plane).

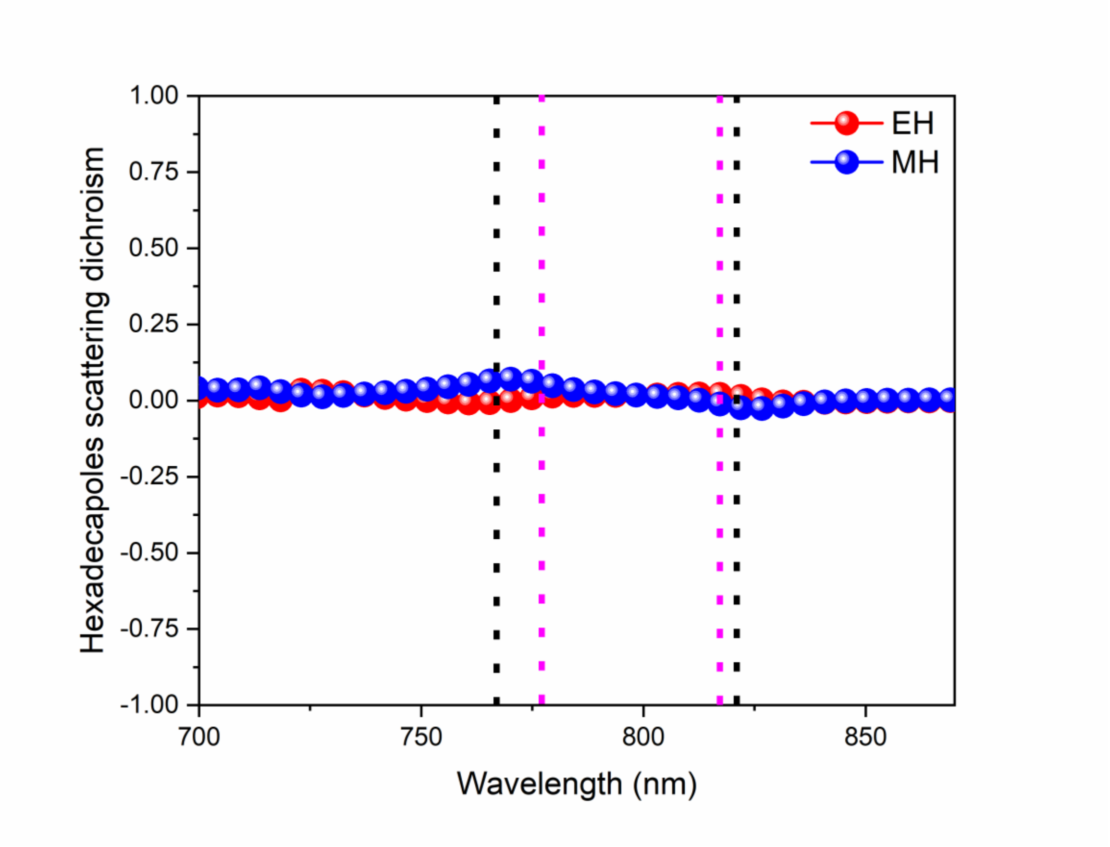

Supplementary Figure 16 | Hexadecapoles scattering dichroism. Contribution of the hexadecapoles scattering dichroism for the left-handed perovskite chiral metasurface.

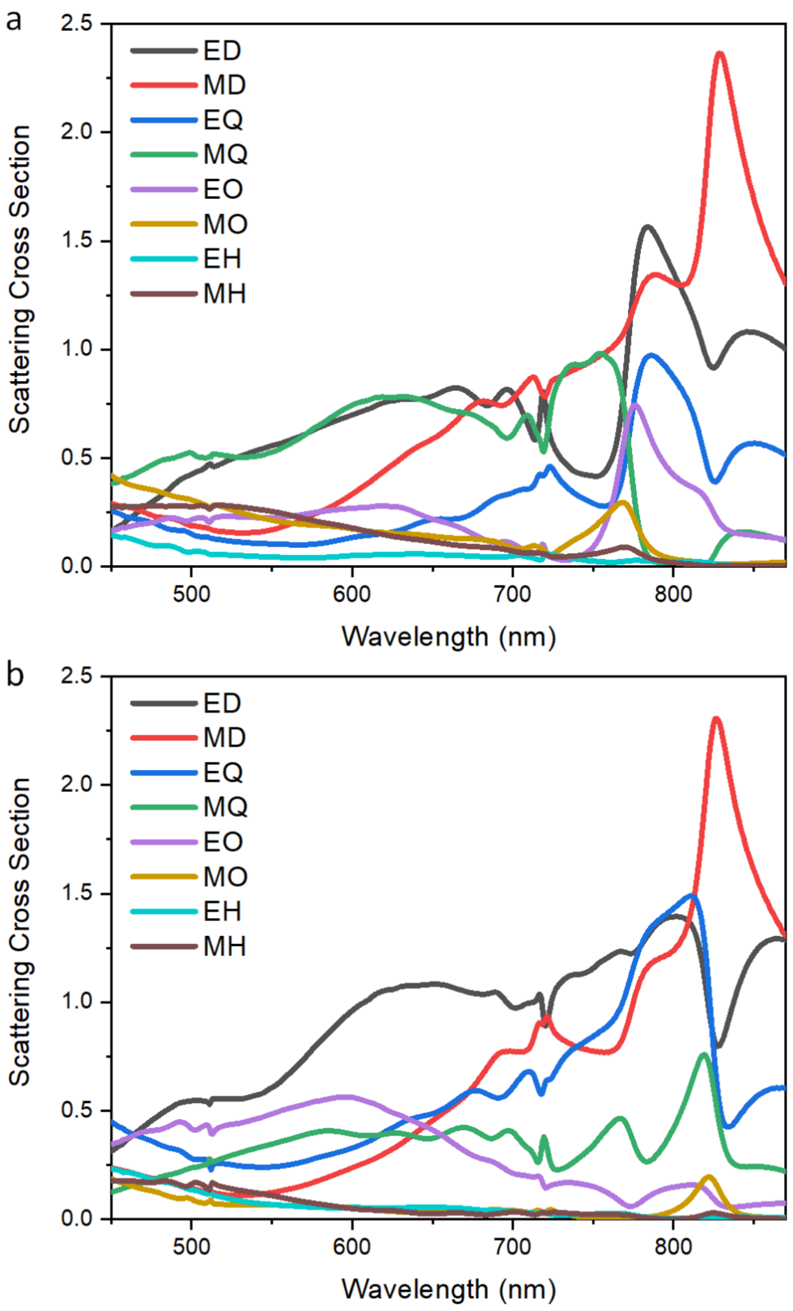

Supplementary Figure 17 | Electromagnetic multipoles of the left-handed perovskite chiral metasurface under LCP (a) and RCP excitation (b). ED is the electric dipole, EQ is the electric quadrupole, EO is the electric octupole, EH is the electric hexadecapole, MD is the magnetic dipole, MQ is the magnetic quadrupole, MO is the magnetic octupole and MH is the magnetic hexadecapole.

Supplementary Note 1

Transmission (T), reflection (R) and absorption (A) of the chiral metasurface under LCP or RCP illumination are given by:

$$T_{\text{LCP}} = \frac{I_{\text{T}}}{I_{\text{in,LCP}}} \quad \text{(Eq. S1)}$$

$$R_{\text{LCP}} = \frac{I_{\text{R}}}{I_{\text{in,LCP}}} \quad \text{(Eq. S2)}$$

$$A_{\text{LCP}} = 1 - T_{\text{LCP}} - R_{\text{LCP}} \quad \text{(Eq. S3)}$$

$$T_{\text{RCP}} = \frac{I_{\text{T}}}{I_{\text{in,RCP}}} \quad \text{(Eq. S4)}$$

$$R_{\text{RCP}} = \frac{I_{\text{R}}}{I_{\text{in,RCP}}} \quad \text{(Eq. S5)}$$

$$A_{\text{RCP}} = 1 - T_{\text{RCP}} - R_{\text{RCP}} \quad \text{(Eq. S6)}$$

where I_T and I_R are the total intensities transmitted and reflected by the metasurface and $I_{in,LCP}$ and $I_{in,RCP}$ are the LCP and RCP input intensities. The circular dichroisms (CDs) of transmission, reflection and absorption are defined as:

$$CD_{\text{T}} = T_{\text{RCP}} - T_{\text{LCP}} \quad \text{(Eq. S7)}$$

$$CD_{\text{R}} = R_{\text{RCP}} - R_{\text{LCP}} \quad \text{(Eq. S8)}$$

$$CD_{\text{A}} = A_{\text{RCP}} - A_{\text{LCP}} = -(CD_{\text{T}} + CD_{\text{R}}) \quad \text{(Eq. S9)}$$

Based on the simulation results, the CD is induced by selective absorption/transmission from the chiral structure, while reflection does not contribute (Supplementary Fig. 5).

Supplementary Note 2

The ellipticity (θ, mdeg) of CD is defined by:

$$\theta(\text{mdeg}) = \frac{180000}{\pi} arctan(\frac{\sqrt{T_{\text{RCP}}}-\sqrt{T_{\text{LCP}}}}{\sqrt{T_{\text{RCP}}}+\sqrt{T_{\text{LCP}}}}). \quad \text{(Eq. S10)}$$

Since $\frac{\sqrt{T_{\text{RCP}}}-\sqrt{T_{\text{LCP}}}}{\sqrt{T_{\text{RCP}}}+\sqrt{T_{\text{LCP}}}} \leq 1$, $arctan\, x \cong x$ and Eq. 10 becomes:

$$\theta(\text{mdeg}) \cong \frac{180000}{\pi} \frac{\sqrt{T_{\text{RCP}}}-\sqrt{T_{\text{LCP}}}}{\sqrt{T_{\text{RCP}}}+\sqrt{T_{\text{LCP}}}} \quad \text{(Eq. S11)}$$

Introducing absorbance and absorbance dichroism:

$$\text{Abs}_{\text{LCP}} = -lg\,\frac{I_{\text{T}}}{I_{\text{in,LCP}}} = -lgT_{\text{LCP}}, \quad \text{(Eq. S12)}$$

$$\text{Abs}_{\text{RCP}} = -lg\,\frac{I_{\text{T}}}{I_{\text{in,RCP}}} = -lgT_{\text{RCP}}, \quad \text{(Eq. S13)}$$

$$\Delta\text{Abs} = \text{Abs}_{\text{RCP}} - \text{Abs}_{\text{LCP}} = -(lgT_{\text{RCP}} - lgT_{\text{LCP}}), \quad \text{(Eq. S14)}$$

Eq. S11 can be rewritten as:

$$\theta(\text{mdeg}) = \frac{180000}{\pi} \frac{e^{\frac{-(ln10)\cdot\text{Abs}_{\text{RCP}}}{2}} - e^{\frac{-(ln10)\cdot\text{Abs}_{\text{LCP}}}{2}}}{e^{\frac{-(ln10)\cdot\text{Abs}_{\text{RCP}}}{2}} + e^{\frac{-(ln10)\cdot\text{Abs}_{\text{LCP}}}{2}}} = \frac{180000}{\pi} \frac{e^{\frac{-(ln10)\cdot\Delta\text{Abs}}{2}} - 1}{e^{\frac{-(ln10)\cdot\Delta\text{Abs}}{2}} + 1}. \quad \text{(Eq. S15)}$$

Since ΔAbs << 1, the exponentials in Eq. S15 can be expanded in Taylor series to first order, and terms of ΔAbs can be discarded in comparison with unity, and the ellipticity finally becomes:

$$\theta(\text{mdeg}) = \Delta\text{Abs} \cdot \left(\frac{\ln 10}{4}\right) \cdot \frac{180000}{\pi} = -(lgT_{\text{RCP}} - lgT_{\text{LCP}}) \cdot \left(\frac{\ln 10}{4}\right) \cdot \frac{180000}{\pi} \quad \text{(Eq. S16)}$$

Correspondingly, the anisotropy factor is given by:

$$g_{\text{CD}} = \frac{2(\text{Abs}_{\text{RCP}} - \text{Abs}_{\text{LCP}})}{\text{Abs}_{\text{RCP}} + \text{Abs}_{\text{LCP}}} = \frac{2(lgT_{\text{RCP}} - lgT_{\text{LCP}})}{lgT_{\text{RCP}} + lgT_{\text{LCP}}} \quad \text{(Eq. S17)}$$

Supplementary Note 3

The FIB fabrication will inevitably induce some damage and Ga contamination of the samples. We analyzed reflection and transmission of areas of $MAPbI_3$ perovskite films on quartz, exposed to FIB milling (exposed area 1, dose=1 mC/cm^2 & exposed area 2, dose=1.3 mC/cm^2). Note that this situation is very far from the actual fabrication condition of metasurfaces, in which the implanted regions of the samples are completely removed.

The two exposed areas show some differences compared to the pristine film, as seen by the optical microscope images (Supplementary Fig. 18a and 18b), due to a combination of:

i) change in thicknesses, as confirmed by AFM measurements (Supplementary Fig. 18c)

ii) a slight modification of both real and imaginary part of the refractive index, n and k (Supplementary Fig. 18d and 18e). The change in refractive index could be attributed to both local heating of the exposed areas, as confirmed by changes of the film roughness and morphology seen in AFM (Supplementary Fig. 18c), and Ga implantation.

Given that the CD simulations of the gammadions using the optical constants of pristine films are in excellent agreement with the results obtained experimentally, we conclude

that the modifications of the film induced by FIB fabrications do not significantly alter the CD of the perovskite metasrufaces.

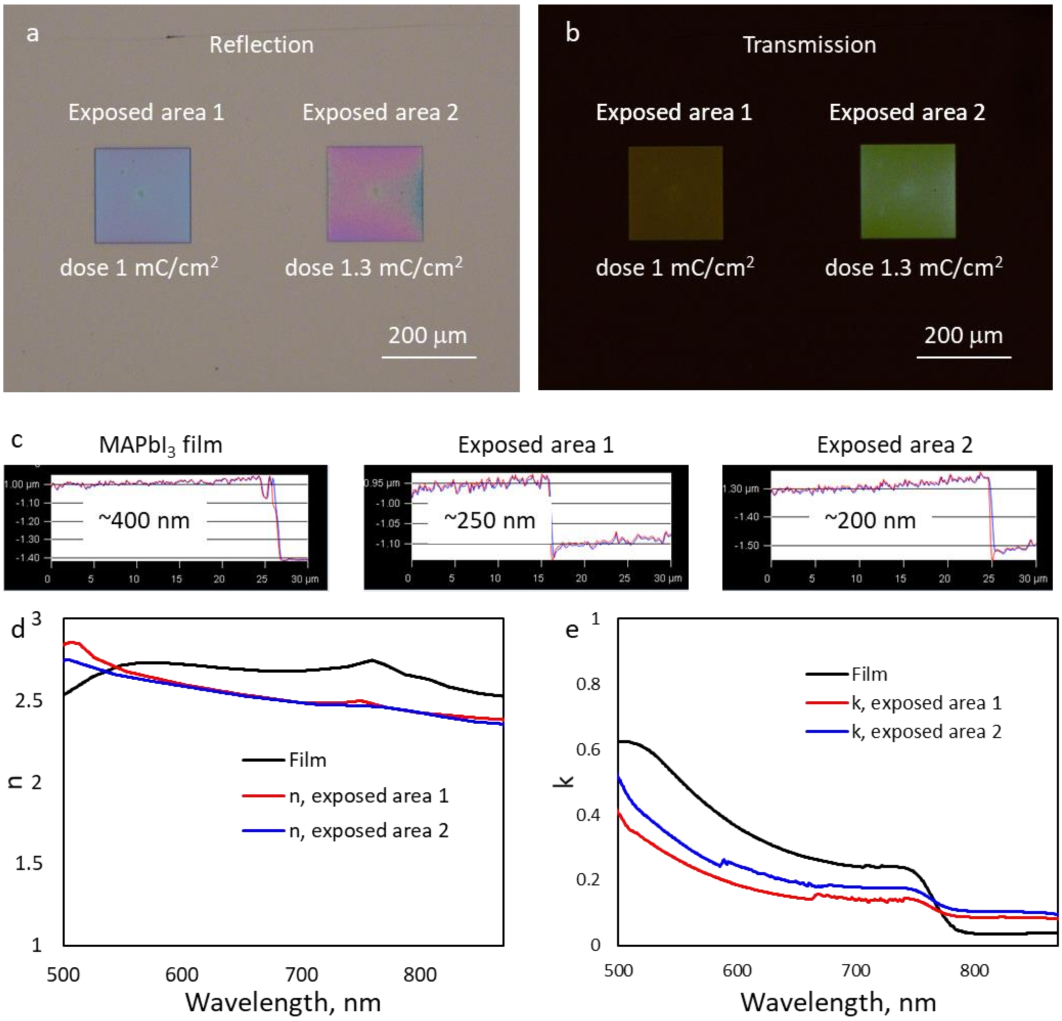

Supplementary Figure 18 | Optical microscope images in reflection (a) and transmission mode (b) of pristine $MAPbI_3$ film on quartz with two 200 µm × 200 µm FIB exposed areas. 1 mC/cm^2 (structure 1) and 1.3 mC/cm^2 (structure 2) dosages were used. (c) AFM measurements of thickness and roughness of pristine $MAPbI_3$ film, structure 1 and structure 2; (d) real and (e) imaginary part of the refractive index of pristine $MAPbI_3$ (black curve), structure 1 (red curve) and structure 2 (blue curve).
